# The use of physical restraints in long-term care in Spain: a multi-center cross-sectional study

**DOI:** 10.1186/s12877-017-0421-8

**Published:** 2017-01-21

**Authors:** Gabriel J. Estévez-Guerra, Emilio Fariña-López, Eduardo Núñez-González, Manuel Gandoy-Crego, Fernando Calvo-Francés, Elizabeth A. Capezuti

**Affiliations:** 1 Nursing Department, University of Las Palmas de Gran Canaria, Tahiche, Spain; 2 Clinic Sciences Department, University of Las Palmas de Gran Canaria, Tahiche, Spain; 3 Nursing Department, University of Santiago de Compostela, Santiago de Compostela, Spain; 4 Nursing Department, University of Las Palmas de Gran Canaria, Las Palmas de Gran Canaria, Spain; 5 William Randolph Hearst Foundation Chair in Gerontology, Hunter College of the City University of New York, New York, NY USA; 6 Unidad Docente de Enfermería de Lanzarote. Facultad de Ciencias de la Salud. ULPGC, C/Rafael Alberti 50, Tahiche, 35507 Spain

**Keywords:** Aged, Long-term care, Restraint, physical

## Abstract

**Background:**

Physical restraint is a procedure used frequently in long-term care. It is a controversial practice because its use is associated with numerous complications and also affects freedom and individual autonomy. The objective of this study was to examine the use of physical restraint of long-term care residents with the ability to move voluntarily.

**Methods:**

We conducted a cross-sectional observational and correlational multi-center study. Nine centers agreed to participate. Of the 1,200 people present at the time of data collection, those without voluntary movement or in the facility for less than a month were excluded. Thus, the final sample was 920 residents. Data on the use of restraints was collected by direct observation. Information about the age, gender, length of stay, falls, mobility, cognition and functional status of residents was gathered by reviewing clinical records and interviewing nursing staff. A descriptive analysis of the data obtained was conducted. The generalized linear model was used, considering only the principal effects of each variable and using the logit link function. The model has been adjusted for clusters and for other possibly confounding factors. For all analyses, a confidence interval (CI) of 95% was estimated.

**Results:**

The prevalence of residents with at least one physical restraint was 84.9% (95% CI: 81.7–88.1), with variability between centers of 70.3 to 96.6% (*p*-value Kruskal Wallis test <0.001). Full-enclosure side rails were most often used (84.5; 95% CI: 81.1–87.9), but other types of restraints were also used frequently. Multivariate analysis showed that the degree of functional impairment increased the probability of the use of restraint. A significant association was also found between restraint use and the impaired cognitive status of residents.

**Conclusions:**

The prevalence was higher than in studies from other countries. The results emphasize the need to improve the training of nursing staff in the care of residents with impairments in functional and cognitive status. The use of alternative devices and nurse consultants need to be evaluated, and the introduction of specific laws considered.

## Background

Physical restraint is used frequently in long-term care, mainly to prevent falls and, to a lesser extent, to reduce behavioural disturbances or avoid interference with patient treatments [1, 2]. Physical restraint is a controversial practice throughout the world [3, 4]. Its use is associated with numerous adverse effects: contractures, incontinence, pressure sores, loss of muscle tone and reduced mobility are among those that have been reported [5]. It also increases the risk of accidents, which, in some cases, have resulted in the death of the patient [6, 7]. Negative psychological effects, such as frustration, fear, loss of dignity, anger, aggression and decline in social interaction, have also been identified [8, 9]. Apart from that, physical restraints are considered a violation of the autonomy and freedom of the individual, and their right to take risks [4].

Some characteristics of the residents, such as their reduced ability to perform the activities of daily living, cognitive impairment or a previous history of falls, are contributing factors for using physical restraint [5]. Attitudes and training of professionals, staffing ratios, legal regulations and routine practices also influence its use [2, 10].

According to international publications, the prevalence in the use of these devices in long-term care varies greatly among countries, ranging from 6 to 68% [1, 2, 11–13]. This large variability could be explained by the different definitions of physical restraint, data collection techniques or characteristics of the facility [14]. The most commonly used devices are full-enclosure side rails, belts and chairs with an attached table [2, 12, 15].

Information regarding the use of physical restraints in Spanish long-term care centers is scarce [16, 17]. This is a moderately aged country with 18.2% of the population 65 years and older. The resident profile is predominantly female, with a mean age of 81 years. In 2014, there were approximately 371,000 long-term care residents, 71% of whom were dependent [18].

Spanish long term care centers comprise a wide range of institutions regarding ownership status, size, resident characteristics and the buildings themselves (eg the design, architectural barriers, dimensions and distribution of the rooms and communal spaces) [18, 19].

There are also significant differences related to personnel ratios, which, on occasion, are insufficient; training is sometimes poor and staff not always sufficiently qualified [20]. The centers are usually public, subsidized or privately owned, with 54% of all these receiving government funding [18]. The study was focused on public centers, as they tend to have similar characteristics.

There is currently no national legislation regarding the use of physical restraint and thus the regulation of this practice is based on regional laws. In spite of this, legal regulations in the Canary Islands are similar to those of other regions. In general, these are undeveloped, allowing the use of restraint to protect the resident from harming himself or others. As a result, its application is at the discretion of professionals [21].

An important issue in prevalence studies is when to determine if a device should be considered a physical restraint. Full-enclosure side rails, for example, can be used to limit the freedom of movement of a person, preventing them from getting out of bed and thus, according to a recent international definition, are physical restraints [22]. Conversely, the use of full-enclosure side rails with patients suffering severe functional impairment who are unable to move voluntarily should not be considered as restraint. Other norms, such as US nursing home regulations, similarly do not consider full-enclosure side rails to be a restraint in these situations [23]. Our intent is to quantify the use of physical restraints and not simply to count the number of devices used. Using the latter method may lead to overestimating the number of devices used and might not give a true count of physical restraint use.

Accordingly, the objective of this study was to examine the prevalence rate of physical restraint of long-term care residents with the ability to move voluntarily. The association of physical restraint with demographic and clinical resident characteristics was also explored.

## Methods

### Study design

A cross-sectional observational and correlational multi-center study was conducted.

### Setting

All the public centers in the Canary Islands, Spain, with more than 80 beds assigned to long-term care were invited, by letter, to participate. All of them accepted, comprising a total of 1,238 beds in 30 units within nine centers. These centers had an average size of 153.5 ± 66.6 (ranging from 88 to 285 beds); one of them had a dementia care unit and two operated a protocol regulating the use of physical restraint. The objectives, methodology and possible benefits of the study were explained to the center managers; a nurse was nominated by each center for the coordination of the study and to facilitate access to patient information. Data were collected between July 2013 and September 2014 due to the geographical dispersion of the islands.

### Participants

The study population consisted of all residents who were present in the centers on the day of data collection. Those residents living in the center for less than a month were excluded from the study, as well as residents with no voluntary movement.

### Variables and data collection

In this study physical restraint was defined as *“Any action or procedure that prevents a person’s free body movement to a position of choice and/or normal access to his/her body by the use of any method that is attached or adjacent to a person’s body and that he/she cannot control or remove easily”* [22].

Information on the use of physical restraint was collected by direct observation [12, 24, 25], as this method seems to be the most reliable [26]. Two trained nurse researchers visited the units on four separate occasions during the same day. The observations were conducted on weekdays at times when residents were either most likely to be active (10:00, 17:00) or when at rest (14:00, 21:00). Visits were determined randomly and not communicated to the facility staff or administrators. Members of the nursing staff accompanied the investigators when the residents were in their own rooms. Prevalence was defined as the percentage of patients with at least one physical restraint documented during any of the observations.

At the end of the study, the validity of the observations between the two investigators was assessed and the inter-rater reliability, using a random sample of 89 residents who were not part of the study, was found to be high (Cohen’s Kappa = 0,977; 95% Confidence Interval [CI]: 0,931 to 1).

These two researchers also examined the clinical records in order to obtain information regarding age, gender, length of stay, falls and cognition of the residents. The registered nurses most knowledgeable of the resident evaluated functional status and mobility. The first of these was determined using the Barthel Index. The overall range could vary between 0 and 100, and the staff’s perception of performance was categorized in different degrees of dependency according to their score: “Total” if <20, “Severe” if between 20 and 35, “Moderate” if between 40 and 55, “Mild” if ≥ 60, and “Independent” if the patient scored 100 (90 if using a wheelchair) [27].

Mobility was measured using the items “activity” and “mobility” of the Braden Scale [28]. Residents’ mobility was graded between 2 points (no mobility) and 8 points (high mobility). Those with less than 4 were not included in the analysis [15]. Cognitive state data were extracted, when available, from the clinical record of each resident. This information was derived from the Mini Mental Status Examination (MMSE) [29]. It was categorized as severe, moderate, mild or intact. All the centers used the version validated by Lobo et al. [30].

The assessment of the reasons that led to the application of restraint was based on a review of the previous six months of clinical records. Given the paucity of these records, this data had to be supplemented with information provided by the registered nurses.

### Sample size

No assumptions were made concerning prevalence of restraint use because of the scarcity of data available about Spain. Usually, an intra-cluster correlation coefficient (ICCC) of between 0.01 and 0.02 is used in human studies [31]; in this work, an ICCC of 0.03 was applied to increase the sample size and decrease its error. As a result, the sample size would be 1,152 individuals (95% CI) in 9 centers. Although 1,200 participants were initially recruited, the final sample, after applying exclusion criteria, comprised 920 residents.

### Data analysis

A descriptive analysis of the data obtained was conducted. Categorical variables were summarized in frequencies; numerical variables in mean and standard deviation. For comparison of distributions, the Wilcoxon test was used because the normality assumption was not met. The relationships for categorical variables were evaluated using the chi-square test and for multiple comparisons the non-parametric Kruskal-Wallis test was used. Prevalence of restraints was adjusted for cluster, estimated the corresponding intra-cluster correlation coefficient (ICCC) and the design factor (DF).

An explanatory model of physical restraints use, quantifying the probable associated factors (odds-ratio/adjusted odds-ratios), was estimated. The generalized linear model was used, considering only the principal effects of each variable and using the logit link function. The model has been adjusted for clusters and for other possibly confounding factors (age and length of stay), although this did not improve the model. For all analyses, a confidence interval (CI) of 95% was estimated.

### Ethical considerations

The protocol was approved by the Ethics Committee for Human Research of the University of Las Palmas de Gran Canaria (CEIH 2013–10). Prior to data collection, verbal consent of the resident, or in cases of cognitive disorder, authorization of the family was obtained. In order to preserve the confidentiality of the data, the names of centers and residents were converted to numerical codes and only the investigators had access to the list, which was stored in a password-protected file.

## Results

Data were obtained from 920 residents in 30 units within the nine centers. Since each resident received 4 observations, there were a total of 3,680 observations.

The mean age of the study participants was 80.1 ± 11.09 years. Most were women (63.22%). Overall, 47.44% of the residents presented with total functional impairment and 41.76% with severe cognitive impairment (Table 1). People who were restrained were older (80.7 v. 76.1 years) and their length of stay in the centers was lower. They showed greater functional and cognitive deterioration than those who were not. They also had less mobility (Table 2).

**Table 1:** Characteristics of residents (*n* = 920)^a^

Variables	
Age (years)	80.1 (11.09)
Length of stay (years)	4.22 (4.95)
Gender	
Women	581 (63.22%)
Men	339 (36.78%)
Mobility^b^	5 (±2.0)
Fall previous 6 month	156 (16.9%)
Dependence of ADL	
Total	436 (47.44%)
Severe	175 (19.04%)
Moderate	156 (16.86%)
Mild	135 (14.69%)
Independent	18 (1.95%)
Cognitive impairment^c^	
Severe	314 (41.76%)
Moderate	213 (28.24%)
Mild	104 (13.92%)
Intact	91 (11.78%)
Non-assessable	31 (4.28%)

^a^Not cluster-adjusted. ^b^Median and interquartile range. ^c^ *n* = 167 missings

**Table 2:** Bivariate relations between the use of physical restraints and characteristics of residents (*n* = 920)^a^

	Not restrained (*n* = 123)	Restrained (*n* = 797)	*p*-value
Age (years)	76.10 (12.52)	80.70 (10.73)	<0.001^d^
Length of stay (years)	6.36 (6.38)	3.88 (4.6)	<0.001^d^
Gender			
Women	55 (44.71%)	526 (66.08%)	<0.001^e^
Men	68 (55.29%)	271 (33.92%)	
Mobility^b^	8 (±2.0)	5 (±2.0)	<0.001^e^
Fall previous 6 month			
Yes	32 (26.0%)	124 (15.5%)	<0.004^e^
No	91 (74.0%)	673 (84.5%)	
Dependence of ADL			
Total	3 (2.44%)	433 (54.39%)	<0.001^f^
Severe	9 (7.31%)	166 (20.85%)	
Moderate	33 (26.83%)	123 (15.32%)	
Mild	62 (50.40%)	73 (9.17%)	
Independent	16 (13.01%)	2 (0.25%)	
Cognitive impairment^c^			
Severe	15 (15.46%)	299 (45.69%)	<0.001^f^
Moderate	28 (28.86%)	185 (28.15%)	
Mild	17 (17.52%)	87 (13.38%)	
Intact	35 (35.05%)	56 (8.30%)	
Non-assessable	3 (3.09%)	28 (4.46%)	

^a^Not cluster-adjusted. ^b^Median and interquartile range. ^c^ *n* = 25 missings not restrained and 143 restrained. ^d^Wilcoxon test. ^e^Chi-square test. ^f^Kruskal-Wallis test

The cluster-adjusted prevalence of residents with at least one physical restraint was 84.9% (95% CI: 81.7–88.1), with variability between centers of 70.27 to 96.55% (*p*-value Kruskal Wallis test <0.001). When full-enclosure side rails were not included, the cluster-adjusted prevalence was 36.6% (95% CI: 33.2–40.0).

Table 3 showed that the devices most used were full-enclosure side rails (84.5; 95% CI: 81.1–87.9), followed by belts in chair (26.9; 95% CI: 23.5–30.3) and belts in bed (9.9; 95% CI: 6.5–13.3).

**Table 3:** Frequency of physical restraints

	Cluster-adjusted prevalence (95% CI)	ICCC	DF
Residents with at least one physical restraint	84.9% (81.7–88.1)	0.124	12.52
Full-enclosure side rails	84.5% (81.1–87.9)	0.137	13.60
Belt in chair	26.9% (23.5–30.3)	0.046	5.23
Belt in bed	9.9% (6.5–13.3)	0.050	5.60
Chair with attached table	6.2% (2.8–9.6)	0.044	5.05
Vest restraint	6.1% (2.7–9.5)	0.045	5.14
Wrist/ankle belt	1.2% (0–4.6)	0.052	5.78
Sleep suits	1.2% (0–4.6)	0.052	5.78

Values are cluster-adjusted percentages and intra-cluster correlation coefficients (ICCC). DF = Design factor

The review of the clinical records and staff interviews confirmed that the major reason for the use of restraint was to prevent falls from a bed or a chair (94.2%). The use of side rails was rarely documented in the clinical notes.

A model that integrates the variables described and the use of the restraints was used. The pattern of odds ratios, adjusted for clusters, indicated that the degree of functional impairment increased the probability of the use of restraint. A significant association was also found between restraint use and the impaired cognitive status of residents (Table 4).

**Table 4:** Explanatory model of physical restraints use (cluster-adjusted)

Factor/variable	AOR (95% CI)	*p*-value
Age (years)	0.01 (0.00–0.04)	0.436
Length of stay (years)	0.001 (0.00–0.06)	0.979
Fall previous 6 mouth (yes/non)	0.49 (0.00–1.30)	0.236
Gender (woman)	0.86 (0.15–1.58)	0.018
Mobility (reference: high mobility = 8)		
4	1.28 (0.17–2.74)	0.084
5	1.11 (0.19–2.41)	0.094
6	0.63 (0.40–1.65)	0.229
7	0.05 (0.92–1.01)	0.926
Dependence of ADL (reference: mild/indep.)		
Moderate	0.88 (0.11–1.65)	0.025
Severe	2.95 (1.75–4.15)	<0.001
Total	4.66 (2.93–6.40)	<0.001
Cognitive impairment (reference: intact)		
Mild	0.88 (0.17–1.93)	0.101
Moderate	1.32 (0.39–2.26)	0.006
Severe	1.52 (0.48–2.55)	0.004

## Discussion

The data showed that the use of physical restraints in the centers studied was higher than other studies using direct observation (84.9%). The highest usages reported in the literature were in Northern Ireland, Canada and Taiwan, with values between 62 and 68% [1, 26, 32], and the lowest in Germany with 26.2% [12]. The Netherlands was situated between them, with a prevalence of around 50% [24, 25].

When the use of full-enclosure side rails was excluded, the prevalence dropped to 36.6%. Compared with other studies using a similar methodology, this rate was higher than those recorded in Canada (33.7%) and Singapore (23.3%) [26, 33], although it was far higher than that recorded in Germany, where belts and other restraints are very rare [12].

There are several possible explanations for these differences. The attitude of nursing staff towards the use of physical restraint is an important contributing factor since it will influence the process of decision-making [3]. While nurses often have negative feelings towards this procedure, in practice they use it whenever they consider it necessary, especially to address patient safety concerns [3, 34–36]. This finding was confirmed by reviewing clinical records and interviewing staff: the risk of falls was the most common justification for the use of restraint, as also found in studies from other countries [1, 2]. However, there is insufficient evidence to support this reasoning and, in fact, it has been found that patients continue to fall despite the restraint [37, 38] and, moreover, their use may increase the risk of serious injury or even death [6, 7, 39].

Another aspect that could be related to the high prevalence is that there is very little training in fall prevention or the management of behavioral and psychological problems for professionals [34, 36, 40], leading them to see restraint as one of the few alternatives available. Previous studies also detected a lack of awareness of the potential complications [34, 36, 40], so that staff may erroneously overestimate the benefits of these devices. Teaching programs addressing these issues, supported by other measures, such as the implementation of technical assistance, changes in institutional culture, or support from a specialist consultant, have been found effective in reducing their usage [16, 41, 42].

The absence of effective national legislation regulating the use of physical restraint in long-term care in Spain [21] could be another factor. In countries where the law significantly limits the use of these devices, Germany for example, the prevalence is much lower than in Spain, and belts are little used [12, 15]. In the USA, the introduction of the Nursing Home Reform Act (OBRA, 1987), and subsequent regulations, reduced the use of restraints, excluding side rails, from more than 30% to less than 10% [43, 44]. At present, some Spanish regions have begun to develop a legal framework to limit the use of restraint, both physical and chemical [21].

In addition to the high prevalence found in this study, the level of restraint use variability among the centers investigated was striking. This result has been observed in other studies [12, 13, 25]. Given that many have similar characteristics, we cannot explain the source of this variability.

It was observed that full-enclosure side rails were the most commonly used restraints, as reported in other studies [1, 2, 12, 13, 15]. Professionals often believe they are essential in the prevention of accidents and in the reduction of potential legal liability [40]. However, there is growing evidence to suggest that these devices may not be so beneficial, especially since they have been associated with fatal injuries [45]. Another important observation made in this study is that their use is not routinely recorded in the patient’s history; this suggests that nursing staff do not consider them as a form of restraint [36], which may help to explain their frequent use. However, full-enclosure side rails, when limiting the freedom of movement of the person, should be considered as a restraint. In other countries with more stringent regulations, documentation, including the rationale for their usage, is mandated [6].

Therefore, we believe that decisions about the use of physical restraint should be based on an individualized assessment that takes into account the benefits and risks, as well as possible damage to the dignity and autonomy of the person [4, 38]. Furthermore, their implementation should be agreed between professionals, residents and family members, documented and regularly reviewed [4].

Regarding the relationship between the variables and the application of physical restraint, the multivariate analysis showed that low functional and cognitive status was associated with the use of restraint; these results are consistent with those reported in the literature [1, 12, 13, 25]. This loss of capacity may mean nurses see residents as fragile and dependent, to be protected from all risk, a perception that would lead to a pro-restraint attitude [36, 46]. But it might also demonstrate that they do not have alternatives to the use of restraint in the care of this group of residents, and have to sacrifice the autonomy of these people for their own security [47]. However, it must be remembered that the limitation of movement that characterizes physical restraint is, in effect, increasing a patient’s disability [37, 48]. For this reason, it is important to provide support and training to nurses when caring for residents with these characteristics, especially those aspects of training that could contribute to reducing the use of these procedures.

### Limitations

The sample size of this study could be considered a limitation as it may have reduced the accuracy of the confidence interval. The data regarding some of the variables was obtained from the clinical records; given that studies in nursing homes have highlighted that not all events are recorded in patient notes [49, 50], it is possible that in some cases information might be missing such as the recording of falls. Other data, such as the cognitive impairment evaluation, must be treated with caution as these did not figure in a large number of patient records.

The decision not to include residents without voluntary movement may also have influenced the results, especially their comparability to other studies that did not follow the same criteria; however, we felt this provided a more representative sample of those in which bilateral full-enclosure side rails, and other devices, would be considered a restraint.

Finally, the data were collected uniquely in the Canary Islands, but it is probable that the results would be similar throughout Spain, given that these types of public institution have similar characteristics and operate under similar legislation regarding the use of physical restraint.

## Conclusions

The prevalence in the use of physical restraint in the facilities studied was higher than those in studies from other countries. This is associated with impairments in functional and cognitive status. The results emphasize the need to provide support and training to staff in the care of these residents. Practices that may favor the routine use of this procedure should be revised to address the underlying issues leading to restraint; nurse consultants and the availability of alternative devices also need to be evaluated in Spain. We believe that these strategies should be supported by specific laws that guide practitioners and institutions to provide care in the least restrictive way possible. The high prevalence of physical restraint use, compared to studies in other countries, will hopefully convince legislators of the need to enact legislation that will restrict usage.

## Abbreviations

ADL: Activities of daily living
AOR: Adjusted odds ratio
CI: Confidence interval
DF: Design factor
eg (exempli gratia): for example
ICCC: Intra-cluster correlation coefficients
MMSE: Mini Mental Status Examination
n: Number of characteristic values
US: United States
